# Hepatitis B e Antigen Seroconversion Is Related with the Function of Dendritic Cells in Chronic Hepatitis B Virus Infection

**DOI:** 10.1155/2014/413952

**Published:** 2014-12-09

**Authors:** Chunjing Lin, Hai Zou, Shumin Wang

**Affiliations:** ^1^Department of Gastroenterology, First Affiliated Hospital of Wenzhou Medical University, Wenzhou, Zhejiang 325000, China; ^2^Department of Hepatology, Sixth Affiliated Hospital of Wenzhou Medical University, Lishui, Zhejiang 323000, China

## Abstract

*Aim*. To investigate the relationship between hepatitis B e antigen seroconversion and the function of dendritic cells (DC) in patients with hepatitis B virus.* Methods*. The peripheral blood mononuclear cells (PBMC) from 21 chronic HBV patients in immune tolerance state, 23 patients in inactive HBsAg carrier state, and 10 healthy HBV-naive blood donors were incubated and induced into DC in presence of granulocyte-macrophage colony stimulating factor (GM-CSF) and interleukin-4 (IL-4), respectively. The expressions of surface markers on DC were detected by flow cytometry, and the stimulatory capacity of DC in allogenic mixed leukocyte reaction (MLR) was tested by CCK-8, and the level of cytokines released by DC was analyzed by enzyme-linked immunosorbent assay (ELISA).* Results*. DC from patients in immune tolerance showed a remarkably lower surface expression of CD80, CD86, and HLA-DR and exhibited an impaired stimulatory capacity in MLR and reduced secretion of IL-12, as compared to the patients in inactive HBsAg carrier state. There was no significant difference between the indicators from the patients in inactive HBsAg carrier state and healthy subjects. There was a significant difference of HBV DNA level between immune tolerance and inactive HBsAg carrier group (*P* < 0.01) and a negative correlation between HBV DNA level and the expressions of dendritic cells in both groups, respectively (*P* = 0.01).* Conclusion*. DC from patients in inactive HBsAg carrier state shows stronger function in comparison with patients in immune tolerance, the expressions of dendritic cells correlate with HBV DNA level, and the function stage of DC may play an important role in HBeAg seroconversion.

## 1. Background

According to statistics, chronic infection with hepatitis B virus (HBV), which poses serious risks to human, currently affects about 350 million people [1–3], and China alone has over 120 million people. Hepatitis B infection is a leading cause of chronic hepatitis, cirrhosis, and hepatocellular carcinoma [4], accounting for 1 million deaths annually. The natural course of chronic HBV infection can be divided into four phases: immune tolerance, immune clearance, low or nonreplication, and reactivation [5, 6]. Inphase 1 patients are HBeAg positive with high HBV replication but minimal liver disease, and phase 3 is the inactive phase during which HBeAg is absent, viral concentrations are low, and there is minimal inflammatory activity in the liver. Although liver disease activities are low in phase 1, 25% of them tend to have liver disease progression through frequency, extent, and severity of hepatitis flares or acute exacerbations in the second immune clearance; in general, early termination of immune tolerance and subsequent HBeAg seroconversion usually confer a favorable outcome, whereas delayed HBeAg seroconversion may accelerate the progression of liver disease.

In the pathogenesis of persistent HBV infection, both the virus and the immune response of the host play a major role. Recently, accumulating reports have shown that the number and the function of dendritic cells are impaired to a certain extent in patients infected with hepatitis B virus [7, 8]. An impaired function of DC may be suggested to account for HBV antigen-presenting and T cells stimulation defect, which may be the cause of chronic hepatitis B virus infection.

Thus, in this study, we investigated the phenotype and function of dendritic cells generated from peripheral blood mononuclear cells (PBMCs) of patients in immune tolerance and low or nonreplication to determine whether there is any difference between them. We hope that these data will be useful in explaining the difference of immune status and level of virus between the patients from two phases.

## 2. Objectives

The objectives are to detect the functional state of dendritic cells from different patients and relate the function and the state of HBV replication.

## 3. Study Design

### 3.1. Subjects

In total, 44 patients with chronic HBV infection were enrolled from the First Affiliated Hospital of Wenzhou Medical College. All the cases were identified according to chronic hepatitis B diagnosis standard (Conference in Xi'an, China, September 2000); patients were positive for hepatitis B surface antigen for at least 6 months; we excluded the patients with other viral hepatitis infections, and anti-HIV were negative. No patient studied had received antiviral treatment before or had any complication such as hepatic cirrhosis and hepatocellular carcinoma (HCC). The 44 patients were divided into two groups: 21 patients were HBeAg positive in immune tolerance (IT) with high serum levels of HBV-DNA, also seropositivity for HBsAg and anti-HBcAbs and normal alanine aminotransferase (ALT) levels; the level of ALT in serum was measured by autoanalyzer (HITACHI 7600-110ISE); the cutoff for the upper limit of normal (ULN) was ALT 50 U/L; 23 patients were anti-HBeAb positive in inactive HBsAg carrier state (ISC) with undetectable or low levels of HBV-DNA and also HBsAg and anti-HBcAb positivity and normal ALT levels. All the patients in group IT and group ISC were analysed for the HBV genotype. The baseline clinical data were shown in Table 1. As controls, 10 healthy HBV-naive blood donors (NC) were assessed. The study protocol was approved by the ethics committee of the author's unit, and written informed consent was obtained from all individuals.

### 3.2. Dendritic Cell Isolation and Culture

Monocyte-derived DCs (MoDCs) were prepared from peripheral blood mononuclear cells (PBMCs) mainly according to previously established protocols [9, 10]. PBMCs were isolated from freshly drawn heparinized whole blood by Ficoll-Hypaque density gradient centrifugation, washed two times, and resuspended at 2 × 10^6^/mL in RPMI 1640 (RPMI 1640 medium was purchased from GIBCO BRL, Gaithersburg, MD, USA). After 3 h incubation at 37°C in 5% CO_2_ in 6-well plates, supernatant was discarded and adherent cells were incubated in RPMI 1640 plus 10% fetal bovine serum overnight. The nonadherent cells were gently removed, RPMI 1640 with 10% fetal bovine serum supplemented with 100 ng/mL of rhGM-CSF (Peprotech, England). And 50 ng/mL of rhIL-4 (Peprotech, England) was added to the wells. Half of the medium was refreshed and cytokine was added at the middle concentration every 2 days. On the 7th day of incubation, 25 ng/mL of rhTNF-*α* (Peprotech, England) was added to the medium, and then the cells were collected on the 9th day.

### 3.3. Morphology of Dendritic Cells

The morphology of dendritic cells was monitored by light microscope.

### 3.4. Flow Cytometry of Surface Markers

On day 9, dendritic cells were harvested and stained with conjugated monoclonal mouse-anti-human antibodies, FITC-anti-CD80, PE-anti-CD86, and PE-anti-HLA-DR (all purchased from Ebioscience, San Diego, USA), for 20 min at room temperature in darkness. Isotype-matched antibodies were used as controls. After washing once with PBS, dendritic cells were fixed in 1% paraformaldehyde and analyzed by flow cytometry.

### 3.5. T Cell Stimulation

After being treated with 25 *μ*g/mL of mitomycin at 37°C for 30 min, dendritic cells were plated at concentrations of 2 × 10^4^, 1 × 10^4^, and 5 × 10^3^ cells per well separately, then mixed, and incubated with nonadherent PBMC from the same healthy person at concentrations of 1 × 10^5^ cells per well in triplicate. The total volume was adjusted to 200 *μ*L/well, and the cells were incubated in RPMI 1640 with 10% FBS for additional 96 h at 37°C in 5% CO_2_, then 20 *μ*L/well tetrazolium salt (CCK-8) (purchased from Tongren, Japan) was added to the medium for 4 h before the end of culture, while T lymphocyte group (without dendritic cells incubated together) and only RPMI 1640 medium group were, respectively, established as negative control group and background group. Absorbance (*A*) was measured by ELX800G (Biotech, USA) at a detection wavelength of 450 nm and a reference wavelength of 630 nm. The results were expressed as stimulation index (SI) calculated by the following formula: stimulation index = (values of the sample − the background values)/(values of the negative control −* the background* values).

### 3.6. Cytokine Secretion by Dendritic Cell

Supernatants from mixed lymphocyte reaction (MLR) were collected on day 4 and IL-12p70 was detected using an enzyme-linked immunosorbent assay (ELISA), which was purchased from R&D Systems (Minneapolis, MN, USA) and following the manufacturer's instruction.

### 3.7. Data Analysis

Data were expressed as mean ± SD. All data were analyzed utilizing SPSS-XII software. Parameters collected with homogeneity of variance between groups were determined by least significant difference test (LSD), while data with heterogeneity of variance were determined by Dennett T3 test. Correlations between HBV DNA level and the expressions of dendritic cells were evaluated using Spearman's correlation coefficient.

## 4. Results

### 4.1. Morphological Characteristics of Dendritic Cells

**Figure 1:**
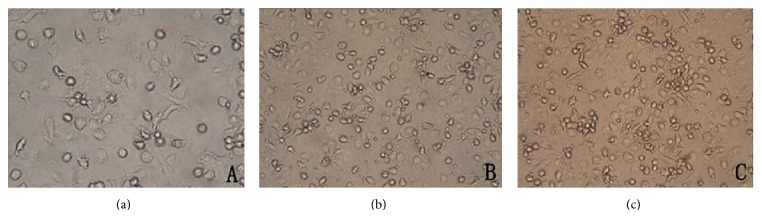
Morphological characteristics of dendritic cells in light microscope that were cultured for 7 days from two kinds of chronic HBV carriers and healthy controls ×200. (a) Dendritic cells cultured from patients in immune tolerance; (b) dendritic cells cultured from patients in inactive HBsAg carrier state; (c) dendritic cells cultured from normal controls.

On the third day, many cells grew branched projections and small adherent aggregates could be observed. On the 7th day, more and more cells were induced and began to suspend. In contrast to another two groups, dendritic cells of patients in immune tolerance have more dead or apoptosis cells with particles in cytoplasm, which are shown in Figure 1.

### 4.2. Phenotype of Dendritic Cells

**Figure 2:**
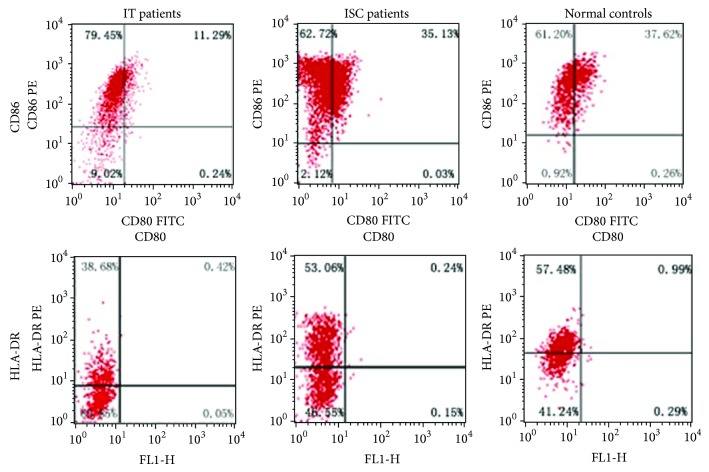
Expression of surface markers on dendritic cells cultured for 9 days.

On day 9 of culture, phenotypic analysis showed that expression levels of CD80, CD86, and HLA-DR were lower in patients in immune tolerance than that in another two groups. Isolated dendritic cells from patients in inactive HBsAg carrier state exhibited similar expression of surface molecules as dendritic cells from healthy controls. The results were shown in Table 2 and Figure 2.

### 4.3. Allogeneic T Cell Proliferation of Dendritic Cells

**Figure 3:**
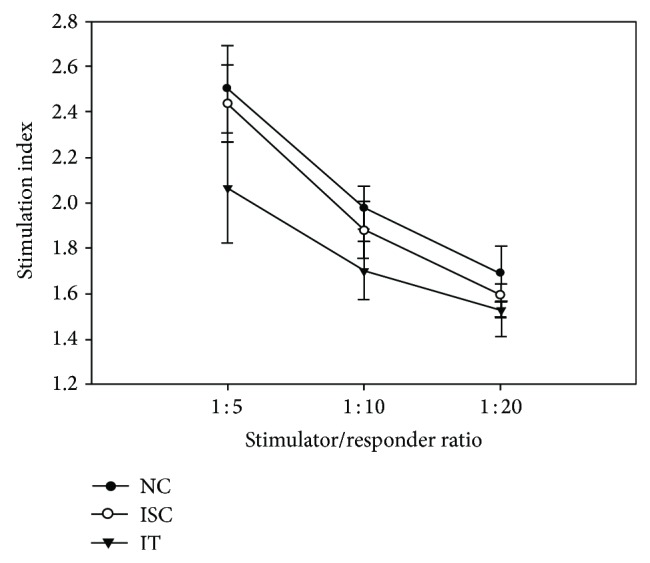
Effect of dendritic cells' stimulation on proliferation of T lymphocytes in mixed lymphocytes reaction.

In allergenic mixed leukocyte reaction, the level of T cell proliferation induced by dendritic cells increased in ratio between DC and T cell-dependent manner. Dendritic cells from ISC patients had a stronger stimulatory capacity than that from IT patients in Figure 3. There was a significant difference between them, especially when DC and T cell were at a ratio of 1 : 5 and 1 : 10. The experiment did not reveal significant differences between DC from ISC patients and healthy control in T cell proliferation.

### 4.4. Cytokine Secretion by Dendritic Cells

The levels of IL-12p70 were 28.11 ± 4.29 pg/mL in IT patients, 34.05 ± 6.11 pg/mL in ISC patients, and 35.46 ± 4.93 pg/mL in healthy controls. The results showed that IL-12p70 was reduced quantitatively in dendritic cells cultured from IT patients compared with ISC patients or healthy controls. However, there were no statistically significant differences between dendritic cells from patients in inactive HBsAg carrier state or healthy people in the secretion of IL-12p70.

### 4.5. HBV DNA Level and Genotype

There was a significant difference of HBV DNA level between immune tolerance and inactive HBsAg carrier group (*P* < 0.01) and a negative correlation between HBV DNA level and the expressions of dendritic cells in both groups, respectively (*P* = 0.01). All the patients were of genotype B or genotype C in these two groups. The percentage of genotype C was higher than genotype B in group IT and group ISC, respectively. Furthermore, the percentage of genotype C in group IT was higher than that in group ISC.

## 5. Discussion

The mechanism of how viral infections evade the immune response and lead to chronic infection has become a research hot spot. It is believed that the cytotoxic T lymphocyte (CTL) response plays a major role in controlling HBV infection [11]. Patients with acute viral hepatitis, who successfully cleared the virus, mounted a large number of HBV-specific CTL response. On the contrary, this response is absent or extremely weak in chronically infected patients who do not clear the virus. Thus, viral persistence or chronicity is associated with an inadequate CTL response [12, 13].

Dendritic cells are considered as the most powerful antigen-presenting cells (APCs) playing a strategic role in initiating and modulating the immune response [14]. DCs are uniquely well equipped in antigen-capturing, processing, and presenting function and act as key players in initiating T lymphocyte activation against viral agents [15, 16]. Beckebaum et al. [17] proposed that HBV infection compromised the antigen-presenting function of DC with concomitant impairment of T helper cell type 1 responses.

In this present study, by the means of GM-CSF and IL-4, DC isolated from IT patients showed decreased expression of CD80, CD86, and HLA-DR and lower allostimulatory capacity when DC and T cell were at a ratio of 1 : 5 and 1 : 10 compared with ISC patients. However, there was no significant difference between them when DC and T cell were at a ratio of 1 : 20. Perhaps the main reason for that is that the number of DCs is too small, leading to no obvious difference in lymphocyte proliferation.

At least, two signals are necessary to activate a naive lymphocyte before it can recognize and target HBV antigen. Binding of the antigen-MHC complex and TCR on naive T lymphocyte represents the first activation signal; costimulatory signal provided by ligation of CD28 by B7 molecules on the naive T cell represents the second signal. Activated DCs have an ability to process antigens and express high levels of costimulatory molecules; thus they can provide both signals needed for T cell activation. In our study, the low expression of costimulatory molecules and reduced allostimulation by IT patients DC would indicate failure of antigen presentation, specially HBeAg presentation and T cell stimulation, inducing lack of HBV-specific immune response, which may play an important role in the difference of immune response between IT patients and ISC patients. By testing the effect of passive immunization with anti-HBe immunoglobulin free of antibody to hepatitis B surface antigen on experimental HBV infection in the chimpanzee model, Stephan et al. [18] suggested that anti-HBe might have biological activity in the modulation of HBV replication. Therefore, the reason for obvious differences in HBV replication between IT and ISC patients may be due to the lack of immune response to HBeAg caused by dysfunction of DC.

The key cytokines provided by DC are considered the third pathway to initiate an adaptive immune response. IL-12 is a main effector in the third pathway to induce helper T lymphocyte (Th) response towards Th1 cell differentiation. It is also involved in the generation of cytotoxic T lymphocyte (CTL) and activation of cytotoxicity in CD8+ T cells, especially potentiating gamma interferon (IFN-*γ*) production by T lymphocytes [19]. Our experimental results showed a reduced secretion of IL-12 by DC in IT patients which induced Th1/Th2 imbalance. This discrepancy may lie in HBV persistent replication.

After HBeAg seroconversion, some patients reach the fourth phase as HBeAg-negative chronic hepatitis B characterized by negative HBeAg, positive anti-HBe, detectable HBV-DNA (10^4^–10^8^ copies/mL), and elevated aminotransferases. There are two hypotheses explaining the reactivation of HBV replication. Precore (Pre-c) mutations abrogate HBeAg synthesis by creating a translational stop codon, while basal core promoter (BCP) mutations reduce HBeAg expression by transcriptional mechanisms [20–22]. Thus, anti-HBe in IT patients is not specific for the mutant virus. This does not seem to be true in HBeAg seroconversion sense. Therefore, no patient in HBeAg-negative chronic hepatitis B was taken into HBeAg seroconversion group.

We found isolated DC from ISC patients exhibited similar expression of surface markers, alloreactive T cell stimulation, and IL-12 secretion as control DC, corresponding to previous findings [23].

HBV genotypes were previously shown to have distinct geographic and ethnic distribution, with genotypes B and C prevailing in Southeast Asia [24], and our present study further confirmed this finding; all the patients were of genotype B or genotype C. Previous studies have shown that HBV genotypes influence disease severity and long-term clinical outcomes of HBV infection [25]. Compared to genotype B patients, genotype C patients have late or absent HBeAg seroconversion after multiple hepatitis flares that accelerate the progression of chronic hepatitis [24, 26]. Most previous studies indicated that patients with HBV genotype C infection have a higher risk of cirrhosis and HCC than those with genotype B infection [27–29]. The impact of viral load on the risk of HCC was assessed in a population-based prospective cohort of untreated CHB Taiwanese patients (REVEAL-HBV study) [30]. We found that the HBV DNA level of genotype C was higher than genotype B. The HBV DNA could influence the expression of dendritic cells concentration. But these differences need to be more strictly reconfirmed by a larger number of cases.

In conclusion, the present study demonstrates that DC shows stronger function after HBeAg seroconversion. The change of the function of DC may play an important role in the difference of immune response and replication of HBV between IT patients and ISC patients. We planned to pulse DC cells with HBeAg or HBcAg to enhance the functions of DC, especially the immune response to HBeAg in further study.

## Figures and Tables

**Table 1:** Clinical characteristics of patients and normal controls (mean ± SD).

Parameters	IT patients (HBV-DNA >10^4^ copies/mL)	ISC patients (HBV-DNA <10^4^ copies/mL)	Normal controls
*n*	21	23	10
Age (years)	30.36 ± 7.79	33.15 ± 6.91	29.10 ± 4.33
Sex (male/female)	10/1	11/2	6/4
ALT (IU/L)	36.45 ± 16.94	29.62 ± 13.91	30.10 ± 11.82
AST (IU/L)	30.64 ± 11.84	29.23 ± 10.03	26.90 ± 12.13
HBV-DNA (lg copies/mL)	6.42 ± 1.07^*^	3.32 ± 3.93	—
Genotype (type B/C)	4/17	9/14	—

Note: ^*^ *P* < 0.01 versus ISC patients.

**Table 2:** Detection of dendritic cells' phenotypes in patients and normal controls (%, mean ± SD).

Group	Case	CD80 (%)	CD86 (%)	HLA-DR (%)
IT patients	21	11.98 ± 6.69^∗#^	90.03 ± 3.01^∗#^	38.40 ± 8.52^∗#^
ISC patients	23	34.83 ± 9.62	95.99 ± 4.59	54.38 ± 12.45
Normal controls	10	37.62 ± 9.88	96.15 ± 3.33	58.32 ± 7.68

Note: ^*^ *P* < 0.01 versus ISC patients and ^#^ *P* < 0.01 versus normal controls.
